# AI, machine learning, and adjudication of hemodynamically significant PDA in extremely premature infants

**DOI:** 10.1038/s41390-026-04828-5

**Published:** 2026-03-20

**Authors:** Danielle R. Rios, Patrick J. McNamara

**Affiliations:** 1 Division of Neonatology, Department of Pediatrics, University of Iowa Stead Family Children’s Hospital, Iowa City, IA USA; 2 Department of Internal Medicine, University of Iowa Health Care, Iowa City, IA USA

Patent ductus arteriosus (PDA) is a common problem in premature infants and is associated with mortality and neonatal morbidity; yet the approach to treatment selection (*who, when, how*) is controversial, with polarized opinions amongst neonatologists. Recent trials of early ibuprofen treatment have shown no benefit in reducing the composite outcome of death and/or bronchopulmonary dysplasia.^1,2^ While patient selection is reflective of real-life practice, with the eligible patient population selection based on PDA diameter >1.5 mm and predominant left-right transductal shunt, treatment was not uniformly effective. The issue of whether the enrolled population is reflective of patients with moderate-high volume shunt is also questionable. The importance of patient selection was demonstrated at a single center where there was no increased risk of untoward outcomes when low-volume PDA shunts, several of which were greater than a diameter of 1.5 mm, for a minimum of 2 weeks, were left untreated.^3^ In addition, when hemodynamic significance is clearly defined, improvements in death, severe intraventricular hemorrhage, and grade 3 bronchopulmonary dysplasia were seen with targeted PDA therapy.^4^ The controversy regarding definition, approach to screening, and management strategies lends itself well to the potential of artificial intelligence and machine learning models in PDA identification and prediction.

**Fig. 1:**
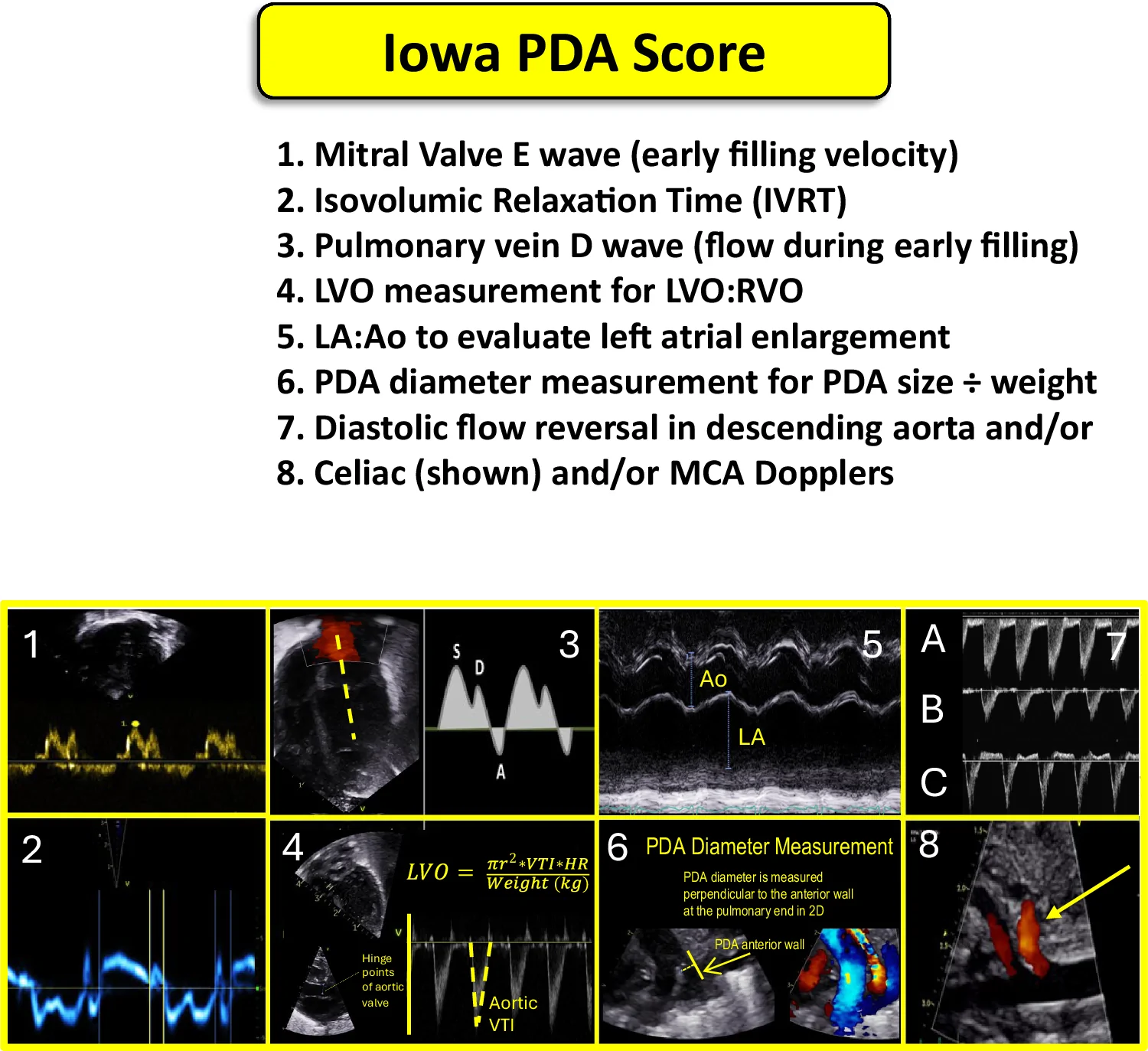
Components of the Iowa PDA Score for hemodynamic significance. Ao aortic root, LA left atrium, LVO left ventricular output, MCA middle cerebral artery, PDA patent ductus arteriosus, RVO right ventricular output.

In this issue of *Pediatric Research*, Kung et al. ^5^ describe a machine learning-based tool to facilitate recognition of premature infants at increased risk of hemodynamically significant PDA (hsPDA). The final model included 6 variables: birth weight, mean fraction of inspired oxygen (FiO_2_) in the first 3 days, mean blood pressure (BP) in first 3 days, percent of time with saturations >96% in the first 3 days, amount of surfactant received in the first 3 days, and mode of delivery. The model achieved a sensitivity of 74.8% and a specificity of 53.4% with an area under the ROC curve of 0.685. The building of this model was based on the assumption that receipt of therapy was pathognomonic of a hsPDA. This approach places a high degree of importance on the validity of the echocardiography definition of hemodynamic significance. In their routine clinical practice, adjudication of hemodynamic significance is based on the presence of a left-to-right PDA shunt, and identification of one of the following echocardiography parameters: end-diastolic flow in the left pulmonary artery >0.2 m/s, diastolic flow reversal in the abdominal aorta/celiac trunk, or left ventricular load (visual estimate of ejection fraction/left atrium to aorta ratio > 1.4/left ventricle end diastolic diameter >15 mm/kg). It has previously been reported that many echocardiography measurements are prone to operator dependent error, influencing their reliability.^6^ Incorrect adjudication of hemodynamic significance may lead to both under- and over-treatment. As an example, echocardiography findings of left ventricular load may also be seen in the setting of LV dysfunction or in patients with systemic hypertension, clinical scenarios where closure of the PDA would not be advised. Medical therapy itself is not without side effects, with animal evidence of impaired angioogenesis;^7^ therefore, avoidance of unnecessary treatment in patients with low volume shunts or where spontaneous closure is likely is paramount. On the other hand, data from the preterm baboon model suggest improved alveologenesis with early ibuprofen.^8^ A comprehensive hemodynamics assessment is, therefore, required to ensure the echocardiography markers are consistent with the findings of hsPDA and not another disease process masquerading as hsPDA, with only one or more markers present.^9^ Screening high-risk patients using a multiparametric scoring system, such as the Iowa PDA score (Fig. 1), distinguishes low-volume shunts, which do not require treatment, from moderate and high-volume shunts, which do, and allows for one or more markers to be present without mistaking the diagnosis as hsPDA when it may be something else entirely.^10^

Routine clinical assessment has been shown to be unreliable in the identification of a hsPDA. The early clinical signs of a high-volume shunt, including increasing oxygen and ventilation requirements, low diastolic and mean BP, and metabolic acidosis, may be attributed to immaturity and other disease states. A prospective observational study of 154 patients showed that only metabolic acidosis consistently predicted the presence of a large PDA on day 7^11^. These data highlight current knowledge gaps and the merits of potential use of machine learning in the identification and prediction of hsPDA, which is a much-needed area of study. The capabilities of machine learning allow for intricate analyses not possible with the commercially available monitoring modalities and current assessment strategies. The authors chose to include only two continuously monitored variables in their final model, which were the average of the mean BP in the first 3 days and percent of time oxygen saturations were >96% in the first 3 days. While these data are valuable, it is also possible that using the average of the mean BP alone over such a long period resulted in a loss of signal that could be delineated with more intricate analyses. In particular, variance in components (systolic vs diastolic BP) and location (pre- vs post-ductal) of BP measurement.^12^ may better distinguish physiologic differences between groups. Additionally, breaking the assessment periods up into smaller chunks for analysis may have resulted in signals to help characterize transductal flow patterns and shunt volume. It is unfortunate that the authors did not have continuous vital sign collection to analyze; however, they did report having values available every 15 min. Another potential improvement in model performance may have resulted from arterial waveform analysis in those with continuous blood pressure monitoring.

One challenge in the creation and implementation of a machine learning model, such as described in the paper, is the reliance on clinical deterioration for identification of hsPDA. It has been shown that findings on echocardiography that identify hsPDA precede clinical signs by up to four days.^13^ Waiting until the infant requires high FiO_2_ to consider assessment for hsPDA using this tool could delay the diagnosis, leading to increased risk for untoward outcomes. Additionally, infants with increasing FiO_2_ requirements are more likely to receive more doses of surfactant therapy, so it is possible that these two variables may be colinear. In addition, the clinical assumption that respiratory deterioration is exclusively of lung origin may result in delays in diagnosis or the administration of biologically counterintuitive or harmful treatments For example, in older preterm infants (more than 6 days old), PDA status is an important determinant of late surfactant response; specifically, infants with a small or closed PDA are more likely to have positive response whereas infants with a PDA ≥ 1 mm were more likely to have a negative response.^14^ In institutions without established screening programs for hemodynamic status, a lack of precision for diagnosis of hypoxemia makes the likelihood of the presence of a hsPDA being treated with surfactant (or other therapy) significantly higher.

While echocardiography provides enhanced diagnostic precision, it is not without limitations. In most hemodynamic centers, it is not routinely available 24/7, which may lead to delays in disease recognition and treatment. Second, the dynamic nature of cardiopulmonary physiology and the developmental vulnerability of immature infants are important considerations, as the underlying phenotype may change significantly and rapidly. For example, inhaled nitric oxide for preterm infants with pulmonary hypertension may lead to major changes in pulmonary vascular resistance and both the direction and magnitude of the ductal shunt. It is difficult to recognize the threshold point of changing phenotype; therefore, the timing of follow-up echocardiography is difficult. Machine learning may facilitate enhanced monitoring and pattern recognition, leading to more accurate phenotype characterization.

Overall, it is commendable that the authors have invested in demystifying the diagnosis of hsPDA in this patient population with a relatively simple tool. It is important to recognize, however, that hsPDA is a complicated diagnosis and simplifying it can lead to both over- and underdiagnosis, resulting in over- or under-treatment. Future research in the area of machine learning and predictive analytics must take into account adjudication of disease by ensuring the ability to distinguish a PDA that is present but does not require treatment (low-volume shunt) from a PDA that requires treatment (moderate or high-volume shunt) from a patient with an absence of PDA. This may require physiologic variables such as continuous vital sign monitoring, waveform analysis, and/or echocardiographic markers of volume loading to accomplish. The focus should remain on early hsPDA identification and precision therapy for the mitigation of adverse outcomes.
